# Intercropping of short- and tall-stature maize decreases lodging risk without yield penalty at high planting density

**DOI:** 10.3389/fpls.2025.1570921

**Published:** 2025-04-30

**Authors:** Jianhong Ren, Dejie Wei, Xinru Zhang, Cai Wu, Wenwen Han, Lingxin Shi, Zhiyi Tang, Zhihua Wu, Guangzhou Liu, Yanhong Cui, Xiong Du, Zhen Gao

**Affiliations:** State Key Laboratory of North China Crop Improvement and Regulation, Key Laboratory of Water-Saving Agriculture in North China, Ministry of Agriculture and Rural Affairs, Key Laboratory of Crop Growth Regulation of Hebei Province, College of Agronomy, Hebei Agricultural University, Baoding, Hebei, China

**Keywords:** intercropping, leaf area, photosynthetically active radiation, land equivalent ratio, yield

## Abstract

High planting density of maize usually results in higher grain yield but also raises the risk of lodging. Cultivar intercropping had been proved to improve yield and stress resistance. Thus, we aimed to coordinate grain yield and lodging resistance of maize under high planting density by intercropping short-stalked Zhengdan958 (ZD) with tall-stalked Xianyu335 (XY). Five planting systems were conducted, i.e. SZD: sole Zhengdan 958 at normal density (7.5 plants m^-2^); SXY and SHXY: sole Xianyu 335 at normal and high density (9.0 plants m^-2^); IND and IHD: normal density ZD intercropped with normal and high density XY, respectively. Land equivalent ratio (LER) averaged to 0.99 and 0.96 in two experimental years, indicating no land use advantage of maize variety intercropping compared to monocultures. The average relative yield (partial LER) of ZD was 0.36-0.42, but that of XY was 0.54-0.63, indicating dominance of tall XY in the intercropping. Yield of intercropped XY per meter row was 13.3% and 17.0% higher than sole XY in two years; however, yield of intercropped ZD in IND and IHD was 16.7% and 25.3% lower than sole ZD in this study, respectively. Compared with IND, IHD did not significantly improve the population yield. The upper leaf area of intercropped XY was greater than sole stand, leading to increased interception of photosynthetically active radiation (PAR). However, the increased leaf area of intercropped XY resulted in reduced PAR for ZD, especially at the middle layer where assimilates are directly transported to the ear. Moreover, decreased superoxide dismutase (SOD) activity and SPAD, increased malondialdehyde content of ear leaf was observed for intercropped ZD, due to shading stress caused by tall XY. The principal component analysis indicated upper and middle leaf area, light interception, and SOD were closely related to grain yield. Lodging rate of sole XY under normal and high density was 4.3% and 22.0% in 2021, but lodging was absent for ZD and intercropped XY, which demonstrated that the lodging resistance of intercropped XY was significantly enhanced. This study presents a strategy to enhance maize lodging resistance without yield penalty or requiring additional inputs.

## 1 Introduction

Increasing maize yield plays a crucial role in meeting the demands of the expanding global human population. Increasing planting density has been proven as a key strategy for boosting grain production (Testa et al., 2016). For instance, in the United States, elevating the average density from 55,000 plants per hectare to 97,500 plants per hectare resulted in maize yields rising from 9.0 to 15.0 t ha^−1^ (Zhao and Wang, 2009). In China, a 5.6% enhancement in maize yield was achieved by elevating the density from 60,000 plants per hectare to 75,000 plants per hectare without additional input (Hou et al., 2020).

Exceeding the optimal planting density can trigger a shade avoidance response, leading to increased plant height and ear height, ultimately causing lodging and yield loss (Jafari et al., 2024). In the North China Plain (NCP), one of the most critical maize production regions in China, the planting density of maize generally does not surpass 90,000 plants ha^–1^ due to limited solar radiation and the high risk of lodging (Liu et al., 2022; Xu et al., 2017). Research has shown that plant growth retardants can significantly enhance the lodging resistance and yield of maize (Gong et al., 2021). However, the effects of using exogenous plant growth retardants depend on the variety, density, growth stage, dosage, and climatic conditions (Ren et al., 2024; Zhao et al., 2021). Planting lodging-resistant cultivars present a simple and practical strategy to mitigate maize lodging, yet these cultivars often exhibit lower yields compared to lodging-susceptible ones due to photosynthate competition between stem strength and kernel growth (Zhang et al., 2023). Additionally, challenges in commercializing mutants with dwarfing alleles in maize persist due to abnormalities in flower sexuality and the extreme dwarf and pleiotropic effects of known mutant alleles (Bortiri and Hake, 2007; Cassani et al., 2009). Bayer recently developed a short maize variety with a high harvest index, but widespread adoption remains a distant goal (Wang et al., 2023). Therefore, the pursuit of a simple approach that coordinates the lodging resistance and grain yield is crucial in high-density maize production.

Intercropping, the practice of cultivating different crops or varieties in the same field, is a straightforward method to adapt to climate change and unforeseen stresses (Brooker et al., 2015). Intercropping has demonstrated favorable outcomes for crop production and has reduced uncertainties for producers, particularly small-scale farmers (Tooker and Frank, 2012). Recently, increased productivity using cultivar intercropping has been found in a wide range of crops and regions. Optimized cultivar combinations in intercropping have been shown to enhance the lodging resistance of winter wheat (Cai et al., 2019; Kong et al., 2022), increase grain yield, and ensure yield stability (Borg et al., 2018; Fletcher et al., 2019; Kong and Zhao, 2023). Wang et al. (2017) indicated that intercropping of different maize varieties can increase the population yield due to border effects. Li et al. (2023b) showed that mixing maize varieties with diverse drought tolerance and flowering traits increased the yields via pollination synchrony. However, maize cultivar mixtures do not always increase the productivity (Burton and Kemanian, 2022; Su et al., 2023), and its effects on lodging were rarely reported.

Intercropping usually alters the homogeneous canopy to heterogeneity, thereby changing the pattern of canopy light interception (Jurik and Van, 2004). Efficient interception and utilization of light are the primary reasons for yield advantage in intercropping (Wang et al., 2021a). Additionally, intercropping with heterogeneous canopy structures can enhance solar radiation capture within the canopy by reducing the amount of light reaching the ground (Wang et al., 2020). The dominant species in intercropping, typically the taller component, experiences lesser competition for resources compared to corresponding monoculture, thus potentially being allowed to increase the planting density and achieve high yields (Wang et al., 2021b). Lower solar radiation in the NCP limited the planting density increasing. Thus, we speculated that maize cultivars intercropping with different plant heights could allow a higher planting density for taller cultivars, ultimately increasing population grain yield. At the same time, lodging risk would be decreased for tall cultivars in an intercropping system, even though under high density, due to decreased competition and shading from neighboring short plants.

A 2-year field experiment was conducted to investigate the impact of maize cultivars intercropping with different plant heights on yield and lodging resistance. We hypothesized that (1) intercropping of short- and tall-stature maize varieties can increase the population yield and increasing the plant density of tall-stalked maize in intercropping can further improve grain yield, (2) the heterogeneous canopy of an intercropping system can optimize light interception, and (3) intercropping of short- and tall-stature maize varieties can enhance maize lodging resistance. This study will provide a new sight of maize cultivation technique for compact planting and lodging resistance.

## 2 Materials and methods

### 2.1 Experimental site

A field experiment was carried over two growing seasons (2020 and 2021) at the Shenzhou Dryland Farming Experimental Station (Hebei Province, China, 37°91′ N, 115°71′ E, elevation: 20 m above sea level). The soil was clay loam, containing 20.1 g kg^−1^ total organic matter, 1.08 g kg^−1^ total nitrogen, 102.5 mg kg^−1^ alkali-hydrolyzable nitrogen, 143.4 mg kg^−1^ available potassium, and 25.8 mg kg^−1^ available phosphorus (Olsen Method). In addition, rainfall and average temperature data on each month for both years were shown in **Supplementary Figure S1**. Total precipitation and average temperature during the summer maize growth period was 507.9 mm, 25.4°C and 736.6 mm, 26.9°C in 2020 and 2021, respectively.

### 2.2 Experimental design

Random blocks design with three replicates was adopted in this experiment. Two commonly used summer maize cultivars with different plant heights were used in this study, i.e., the comparatively long-stalked Xianyu 335 (XY) with a potential plant height of 286 cm and the short-stalked Zhengdan 958 (ZD) with a potential plant height of 246 cm. For taller XY, two plant densities were set up, i.e., normal density (ND; 7.5 plants m^-2^) and high density (HD; 9.0 plants m^-2^; Luo et al., 2023; Tang et al., 2022), and only normal density for ZD. ZD was respectively intercropped with normal and high density of XY in 2:2 row configuration. Thus, five experimental treatments were set up, i.e., sole cropping of normal and high density XY (SXY and SHXY), sole cropping of normal density ZD (SZD), intercropping of XY and ZD under normal density (IND), and intercropping of high density XY and normal density ZD (IHD) (**Supplementary Figure S2**). The plot size was 7 m × 12 m in all plots. The row spacing of XY and ZD was 60 cm in both monoculture and intercrop systems. The distance between adjacent XY and ZD rows was 60 cm in the intercrop. Two maize cultivars were sown on June 13, 2020 and June 15, 2021 at two seeds per seeding hole and were thinned to one plant per hill when the third leaf collar was visible. Compound fertilizer (600 kg ha^-1^, N/P_2_O_5_/K_2_O = 25:8:12) was applied at sowing 5 cm away from the crop rows. Moreover, 750 m^3^ ha^-1^ of irrigation was carried in time after sowing to ensure seedling emergence in 2020 and 2021. Disease, weed, and pests were controlled in all plots following local practice.

### 2.3 Sampling and measurements

#### 2.3.1 Aboveground dry matter, plant height, and leaf area index

Three plants in monoculture and three plants for each cultivar in intercropping were randomly selected at silking and maturity to determine the plant height and ear height. The area of each green leaf was calculated by multiplying the leaf length, width, and the shape factor (0.75) (Gao et al., 2017). Leaf area index (LAI) was the total green leaf area per unit area. The maize plants were then separated into stems, leaves, and ears and oven-dried at 80°C to a constant weight for dry matter weight determination.

#### 2.3.2 Photosynthetically active radiation

At silking in 2020, photosynthetically active radiation (PAR) was measured by using a light meter (LI-250A, Li-Cor Biosciences, Lincoln, Nebraska, USA) above the whole canopy (PAR_a_), below the ear leaf, and at the ground level, respectively. In 2021, PAR was measured at four positions, i.e., above the whole canopy, above the first leaf-above ear, below the first leaf-below ear, and at the ground level, respectively. Measurements were carried out in the middle of each cultivar strip in the intercropping and in the center of monoculture plots. The light transmission ratio (LTR) was calculated as follows:


$$\text{LTR }\left(\%\right)\;=\text{ PA}{\text{R}}_{i}/\text{PA}{\text{R}}_{a}\times \;100$$

“i” indicates a different measuring position within the canopy.

#### 2.3.3 Bending strength and rind penetration strength

At 30 days after silking in 2021, three plants of each cultivar were selected to determine bending strength and rind penetration strength (RPS) using a Stalk Strength Tester (ELK-300 N, Zhejiang, China). In intercropping, two cultivars were separately determined. The tester was oriented vertically toward the ear internode until the stem became parallel to the ground. The highest value achieved during this process was documented as the bending strength. The test probe was inserted perpendicularly into the middle of the internode (basal third internode) at a consistent and gradual pace. The maximum force required to penetrate the stalk epidermis was recorded as RPS. The stalk lodging-resistant index (SLRI) was calculated according to Qian et al. (2024):


SLRI (%) = bending strength/height of gravity center × 100

#### 2.3.4 Soluble sugar and starch

The stem samples taken at silking stage were used to measure soluble sugar and starch content with anthrone-sulfuric acid colorimetry method. Each sample (100 mg) was extracted in 6 mL of distilled water by boiling water bath for 30 min. After cooling to room temperature, the samples were centrifuged twice at 2,000 *g* for 15 min. The supernatants were then transferred to a 50-mL volumetric flask and diluted with distilled water to volume up for soluble sugar content determination. Subsequently, starch within the insoluble precipitate was treated with HCl in boiling water and then neutralized with NaOH. The solutions of soluble sugars and starch were separately analyzed at 625 and 620 nm by using a microplate reader (Epoch 2 Microplate Spectrophotometer, BioTek Corporation, Vermont, USA).

#### 2.3.5 Relative chlorophyll content (SPAD value)

At silking and at 15, 30, and 45 days after silking, three representative plants of each cultivar in each plot were selected. A total of 10 points was evenly selected on the ear leaves (avoiding the veins) to measure relative chlorophyll content by SPAD-502 (Minolta Camera Co., Osaka, Japan).

#### 2.3.6 Superoxide dismutase activity and malondialdehyde content

At 10, 20, 30, and 40 days after silking, fresh ear leaves of each cultivar were sampled with three replicates for each treatment. Moreover, 0.1 g of fresh leaf samples was added to an extraction buffer and homogenized in an ice bath. The homogenate was then centrifuged at 10,000 *g* for 10 min at 4°C. The supernatant was collected as crude enzyme extract. The activity of superoxide dismutase (SOD) was measured according to nitro blue tetrazolium (NBT) photoreduction method, and the content of malondialdehyde (MDA) was assayed using the thiobarbituric acid method (Quan et al., 2004).

#### 2.3.7 Grain yield and land equivalent ratio

At physiological maturity, a sample area of 6 m^2^ (5 m long × 2 rows wide) in the center of a monoculture plot was hand-harvested to determine the final yield. In intercropping, a sample area of 12 m^2^ (5 m long × 4 rows wide), including one strip of each cultivar, was measured. After counting the number of ears, 10 representative ears for each cultivar in each plot were selected to determine the grain number per ear. After threshing, grain moisture content was determined by oven drying method. Grain yield was expressed at 14% moisture content. In 2021, lodging occurred in sole XY plots; all of the ears from lodging maize plants were also harvested to calculate the final yield with weighted average method.

Land equivalent ratio (LER) was calculated as an indicator for land productivity:


$$\text{LER}={\text{pLER}}_{\text{ZD}}+{\text{pLER}}_{\text{XY}}=\frac{{\text{Y}}_{\mathrm{int},\text{ZD}}}{{\text{Y}}_{\text{mono,ZD}}}+\frac{{\text{Y}}_{\text{int,XY}}}{{\text{Y}}_{\text{mono,XY}}}$$

where Y_int,ZD_, Y_int,XY_, Y_mono,ZD_, and Y_mono,XY_ are yields of ZD and XY in intercropping and monoculture. pLER_ZD_ and pLER_XY_ are partial LER (relative yield) for each cultivar. The LER is the relative area needed in monoculture to obtain the same yield as that obtained in a unit area of the intercropping. LER >1.0 indicates that intercropping uses land more efficiently than monoculture, while LER <1.0 means that intercropping has no land use advantage.

### 2.4 Statistical analysis

Statistical analyses were carried out with SPSS 22.0 (SPSS Inc., Chicago, IL, USA). The univariate general linear model was used to assess the effects of different planting systems on population yield and leaf area index. Mean values were compared using Tukey test (*P* < 0.05). The differences between different planting densities in the same planting pattern (that is, SXY and SHXY, INDxy and IHDxy, and INDzd and IHDzd) and differences between different cropping patterns for the same variety (that is, sole stand and intercropping) were compared using a two-sided Student’s *t*-test at *P* < 0.05. Shapiro–Wilk test was employed to evaluate the data normality, and Levene test was used to assess the homogeneity of variances before conducting an analysis of variance and *t*-test. Principal component analysis (PCA) was performed by using Origin 2021.

## 3 Results

### 3.1 Grain yield, LER, and yield components

**Figure 1:**
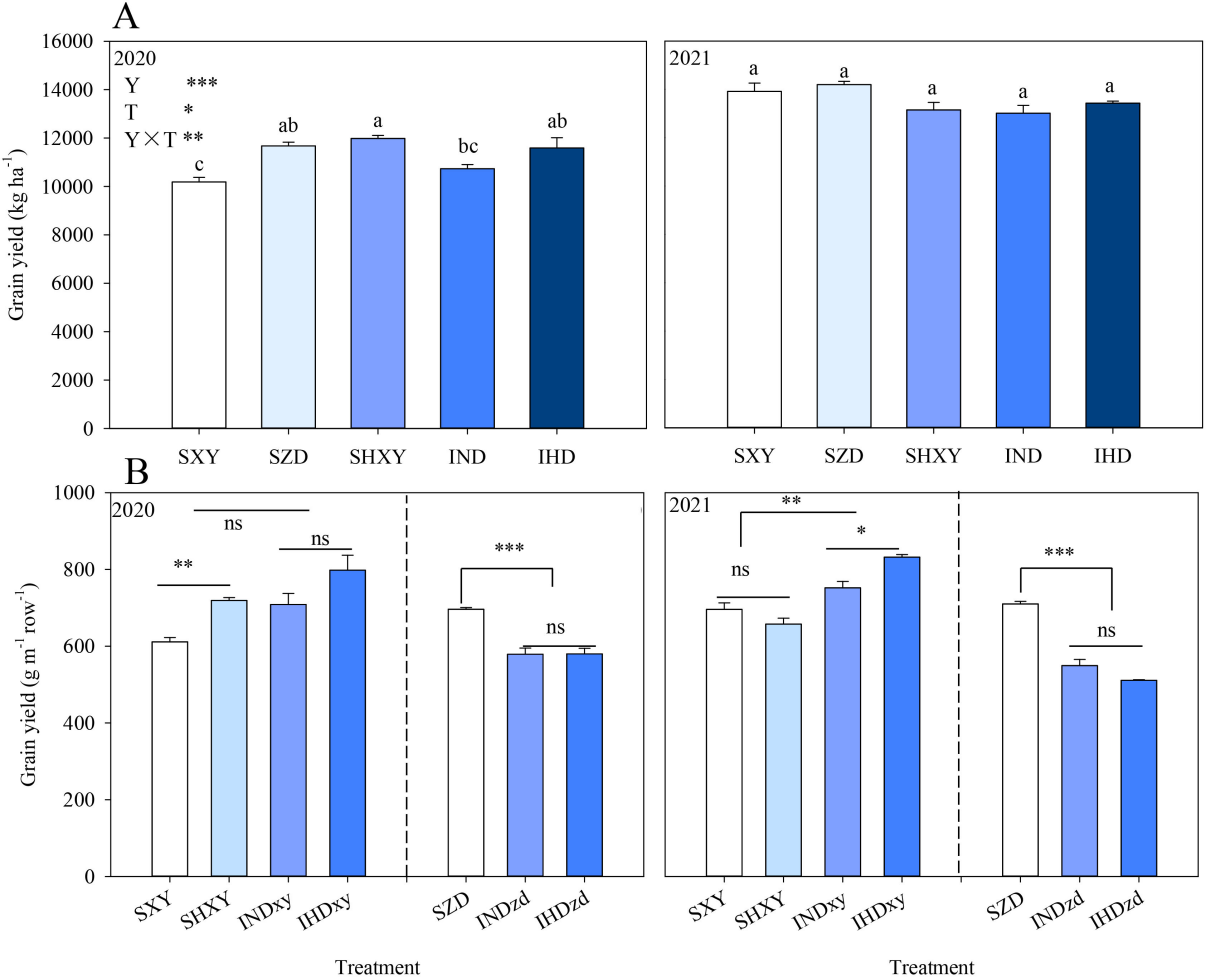
Effects of intercropping of tall- and short-stature maize cultivars on population yield **(A)** and grain yield of each cultivar in per meter row **(B)**. SXY, sole XY335; SZD, sole ZD958; SHXY, sole XY335 with high density; IND, intercropping of ZD958 and XY335 under normal density; IHD, intercropping of normal density ZD958 and high density XY335. Different lowercase letters above bars indicate significant difference among treatments at 0.05 significance level. In **(B)**, xy and zd indicate XY335 and ZD958 in the intercropping system. ns means no significant difference between cropping patterns or planting densities; *, **, and *** indicate significant differences at *p* < 0.05, *p* < 0.01, and *p* < 0.001, respectively.

In two growing seasons, the intercropping systems (IND and IHD) had a comparable yield level with the corresponding monoculture. Increasing the density of high-stalked XY in intercropping marginally improved the population yield by 8.0% and 3.2% in 2020 and 2021 compared with IND, respectively. In 2020, the yield of sole XY was significantly increased under high planting density compared to normal density but experienced a slight decrease in 2021 due to a 22.0% lodging rate (**Figure 1A**). Then, grain yield per meter row was used to further analyze the border row effect of intercropping on two maize cultivars (**Figure 1B**). Year (Y), density (D), intercropping (I), Y × D, and D × I significantly affected the grain yield of XY, while Y, I, and Y × I significantly affected the grain yield of ZD. Averaged over two experimental years, the yield of intercropped XY per meter row in IND and IHD was 12.0% and 18.8% higher than sole XY; however, the yield of intercropped ZD in IND and IHD was 19.7% and 22.4% lower than sole ZD, respectively. Interestingly, intercropped XY under normal density had a similar yield per meter row with high density sole XY.

**Figure 2:**
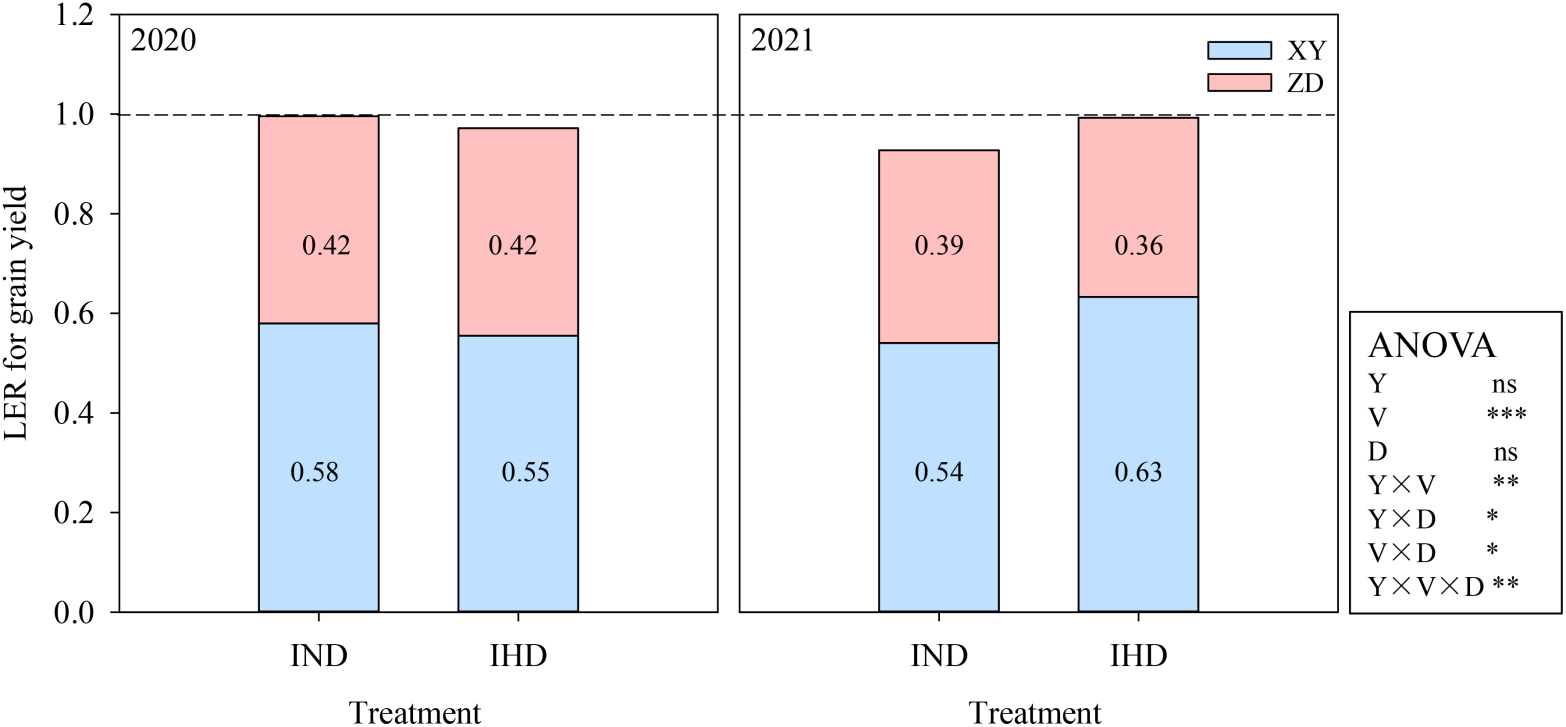
Land equivalent ratio (LER) of grain yield of two intercropping systems in 2020 and 2021. IND, intercropping of ZD958 (ZD) and XY335 (XY) under normal density; IHD, intercropping of normal density ZD958 and high density XY335. Y, year; V, variety; D, density; ns, not significant at 0.05 level; *, **, and *** indicate significance at 0.05, 0.01, and 0.001 level.

XY had yield advantage in intercropping, which had greater pLER (greater than the relative density of 0.5) regardless of planting density in both years. The variety and all the interactions showed significant effects on pLER. Specifically, the pLER of high density XY in IHD reached 0.63 in 2021, which was attributed to the large lodging rate of high density sole XY. However, increasing the density of high-stalked XY in intercropping did not provide a significant yield advantage over normal-density intercropping. Overall, the LERs of two intercroppings were approximately 1, indicating that intercropping with different cultivars did not confer a significant yield advantage compared to sole cropping systems (**Figure 2**).

**Figure 3:**
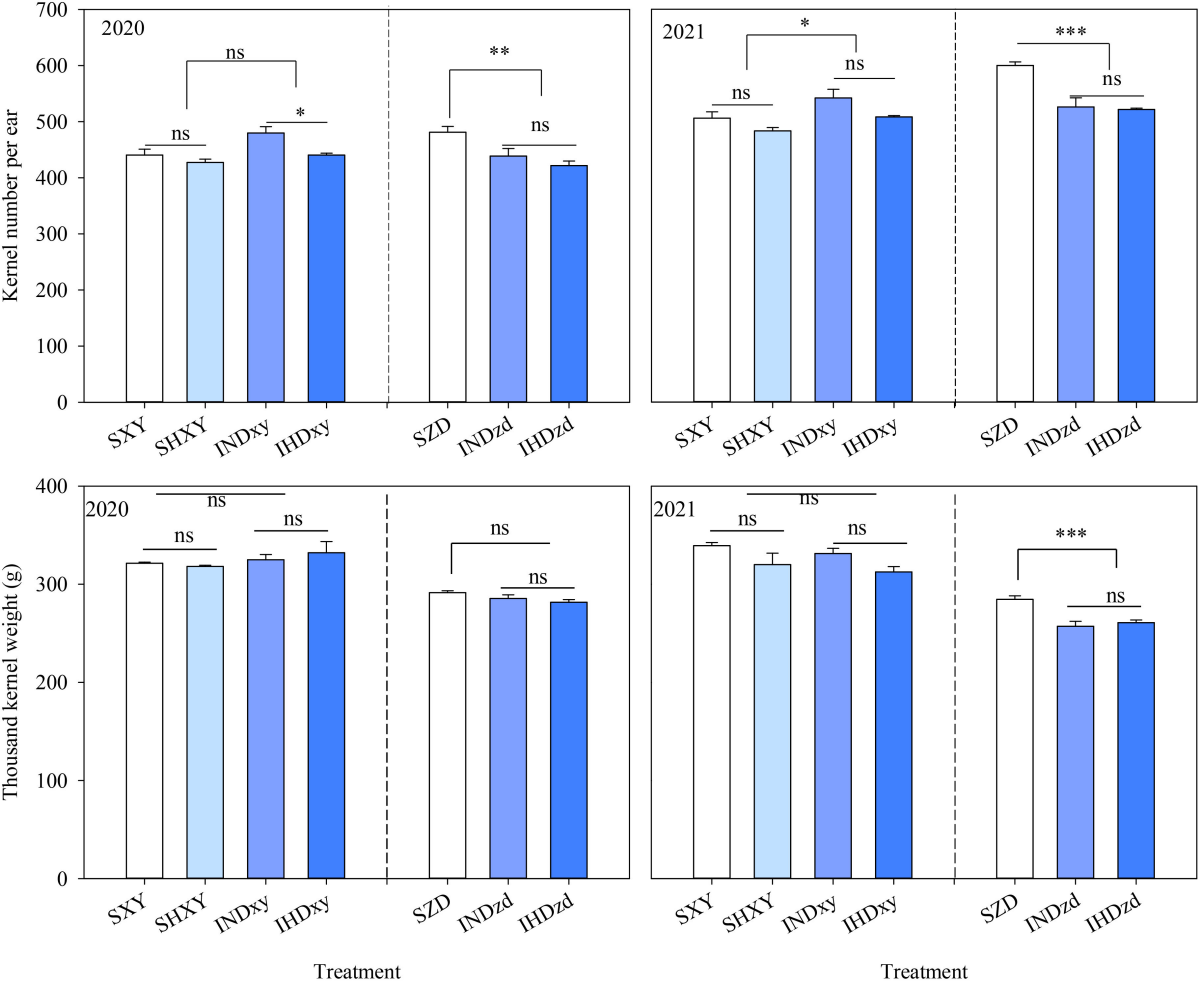
Effects of intercropping of tall- and short-stature maize cultivars on kernel number per ear (KNE) and thousand kernels weight (TKW). SXY, sole XY335; SZD, sole ZD958; SHXY, sole XY335 with high density; IND, intercropping of ZD958 and XY335 under normal density; IHD, intercropping of normal density ZD958 and high density XY335. ns means no significant difference between cropping patterns or planting densities; *, **, and *** indicate significant differences at *p* < 0.05, *p* < 0.01, and *p* < 0.001, respectively.

Y, D, and I significantly affected the kernel number per ear (KNE) of XY. In comparison to sole XY, the KNE of XY in intercropping was averagely increased by 6.0% and 6.1% in 2020 and 2021, respectively. However, intercropping significantly decreased the KNE of ZD over 2 years. The KNE of intercropped ZD was further decreased when intercropped with high density XY, but with no significant difference (*P* > 0.05). Increasing the planting density had no significant effect on the KNE of sole XY in both years. However, the KNE of intercropped XY was significantly decreased under high planting density in 2020. The thousand kernel weight (TKW) of XY was not affected by plant density and cropping system in 2 years. Nevertheless, intercropping markedly decreased the TKW of ZD on average by 9.0% compared to sole ZD in 2021 (**Figure 3**).

### 3.2 Plant height, ear height, and LAI

Intercropping did not affect the plant height or ear height of two cultivars. Increasing the planting density significantly enhanced the plant height of sole XY and intercropped ZD in 2020. The planting density did not affect the ear height of two cultivars, neither in monoculture nor in intercropping. Only Y significantly affected the plant height and ear height. In 2020, the plant and ear height of XY ranged from 253 to 263 cm and from 102 to 108 cm, while those of ZD measured 218–227 and 111–114 cm. In 2021, the plant height for XY and ZD was 310–320 and 285–290 cm, respectively, and ear height that ranged from 128 to 135 cm for XY and 142 to 153 cm for ZD. Notably, XY showed a higher plant height compared to ZD but had a relatively lower ear position (**Supplementary Figure S3**).

**Figure 4:**
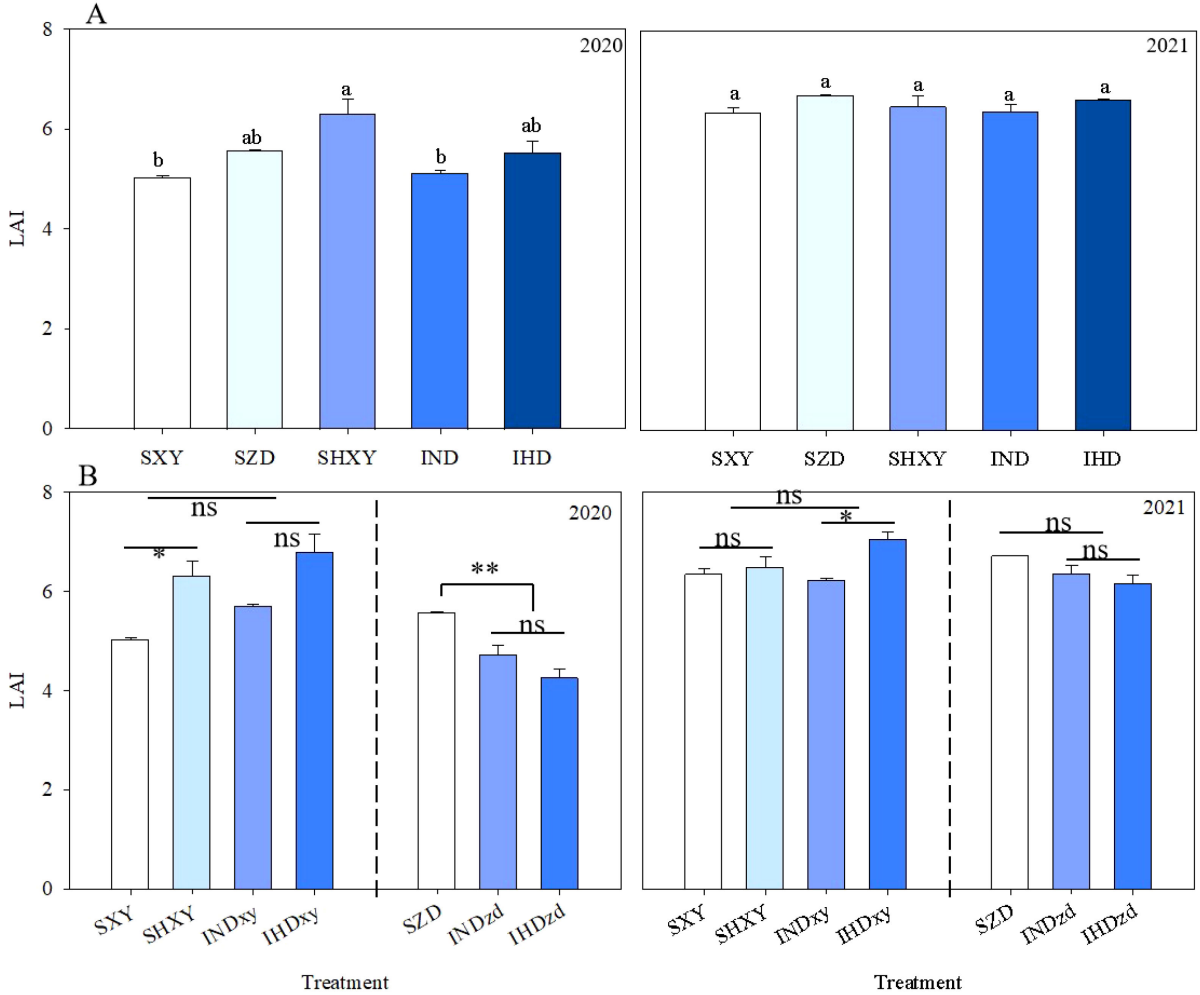
Effects of intercropping of tall- and short-stature maize cultivars on population leaf area index (LAI) **(A)** and LAI of each cultivar in per meter row **(B)**. SXY, sole XY335; SZD, sole ZD958; SHXY, sole XY335 with high density; IND, intercropping of ZD958 and XY335 under normal density; IHD, intercropping of normal density ZD958 and high density XY335. Different lowercase letters above bars indicate significant difference among treatments at 0.05 significance level. ns means no significant difference between cropping patterns or planting densities; * and ** indicate significant differences at *p* < 0.05 and *p* < 0.01, respectively.

No significant differences were observed in leaf area index (LAI) of population among intercropping and corresponding monoculture in 2020 and 2021 (**Figure 4A**). Increasing the planting density of high-stalked XY increased the population LAI, but it was not significant. To further analyze the effect of intercropping on LAI of different cultivars, LAI of per row was determined (**Figure 4B**). Y and D significantly affected the LAI (per row). Intercropping did not affect LAI (per row) of XY in 2 years but markedly decreased the LAI (per row) of ZD by 19.2% in 2020. Increasing the planting density obviously improved the LAI (per row) of sole XY by 25.1% in 2020 and that of intercropped XY by 13.3% in 2021.

**Figure 5:**
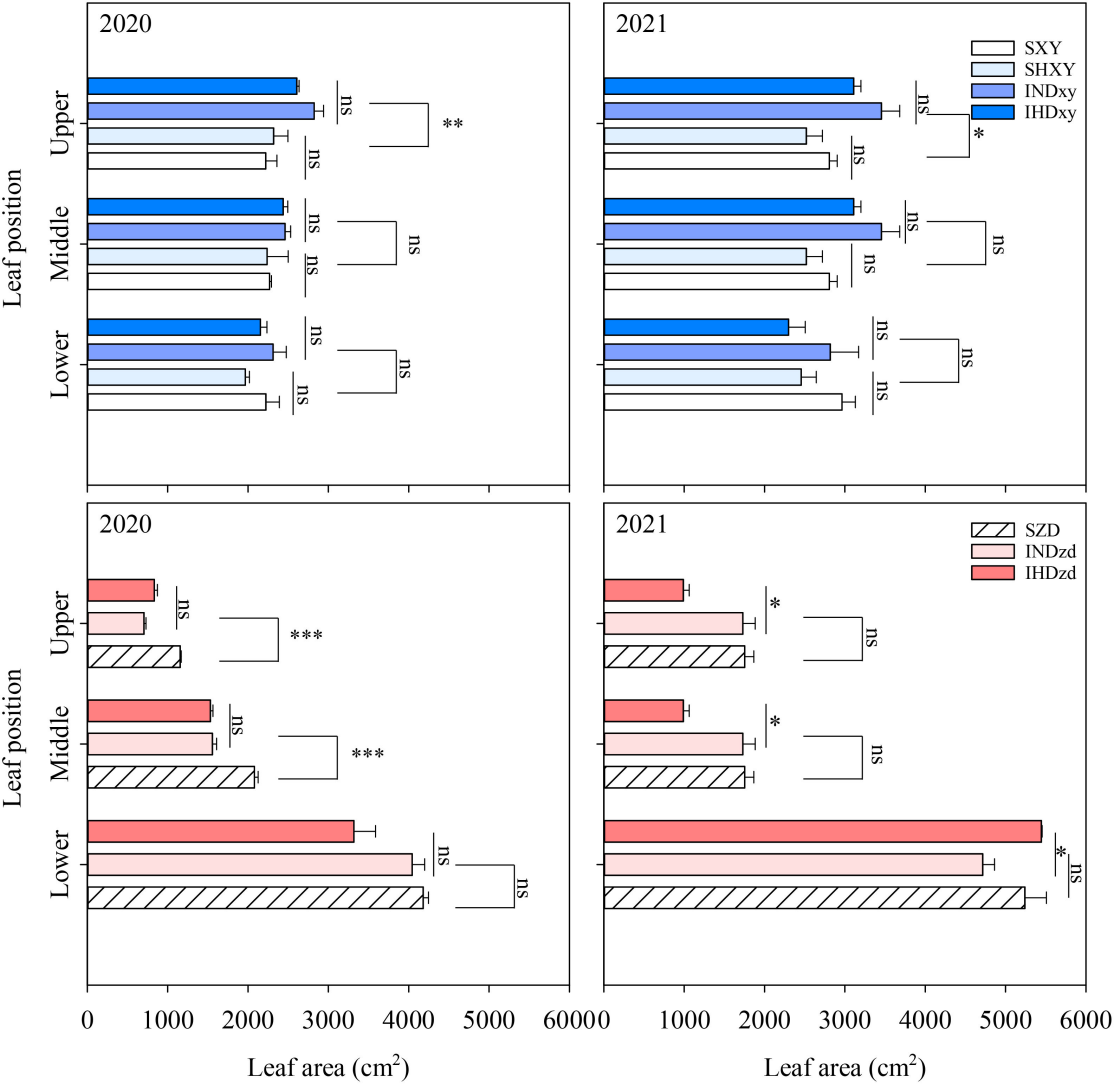
Effects of intercropping of tall- and short-stature maize cultivars on leaf area. The middle layer includes ear leaf, the first leaf above the ear, and the first leaf below the ear. The upper and lower layers indicate the leaves above and below the middle layer, respectively. SXY, sole XY335; SZD, sole ZD958; SHXY, sole XY335 with high density; IND, intercropping of ZD958 and XY335 under normal density; IHD, intercropping of normal density ZD958 and high density XY335. ns means no significant difference between cropping patterns or planting densities; * and ** indicate significant differences at *p* < 0.05 and *p* < 0.01, respectively.

Intercropping significantly affect the upper leaf area of ZD and XY. Across all planting densities, intercropping obviously increased the upper leaves area of XY by 19.8% and 23.2% compared with sole stands in 2020 and 2021, respectively. However, intercropping did not affect the middle and lower leaf areas of XY. In contrast, intercropping significantly reduced the leaf areas of ZD in the upper and middle position by 39.1% and 25.8% compared with sole ZD in 2020, respectively (**Figure 5**). Compared to sole ZD, intercropping had no significant effect on the leaf area of different positions in 2021. The upper and middle leaf areas of ZD were markedly lower than XY, whereas the leaf area of the lower layer of ZD was obviously greater than that of XY (**Figure 5**).

### 3.3 Photosynthetically active radiation and biomass accumulation

**Figure 6:**
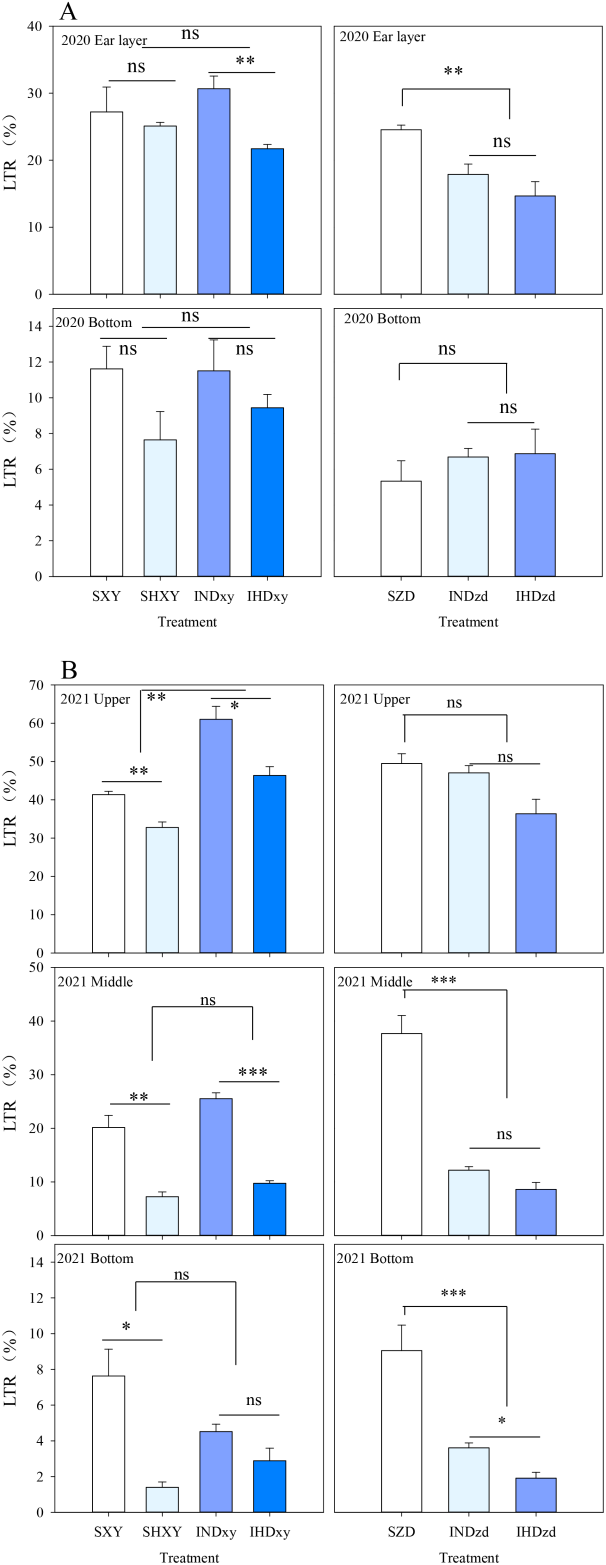
Effects of intercropping of tall- and short-stature maize cultivars on light transmission rate (LTR) in 2020 and 2021. SXY, sole XY335; SZD, sole ZD958; SHXY, sole XY335 with high density; IND, intercropping of ZD958 and XY335 under normal density; IHD, intercropping of normal density ZD958 and high density XY335. ns means no significant difference between cropping patterns or planting densities; * and ** indicate significant differences at *p* < 0.05 and *p* < 0.01, respectively.

In 2020, intercropping did not significantly influence the light transmission ratio (LTR) of tall-stature XY at ear layer or ground level. However, the LTR of intercropped ZD at ear layer was significantly reduced by 33.7% compared to that of monoculture. In other words, light interception above the ear of short-stalked ZD in intercropping was reduced by tall-stalked XY. Additionally, increasing the planting density obviously reduced the LTR of intercropped XY at ear layer (**Figure 6A**). In 2021, intercropping significantly increased the light transmission ratio of XY at the upper canopy by 44.5% compared with sole XY stands but did not affect the LTR of other positions, whereas the LTR of intercropped ZD was significantly reduced by 72.4% and 69.5% compared with sole ZD at the middle and bottom position, respectively (**Figure 6B**). For tall XY, increasing the density significantly decreased the LTR of different positions, except the LTR of intercropped XY at ground level.

**Figure 7:**
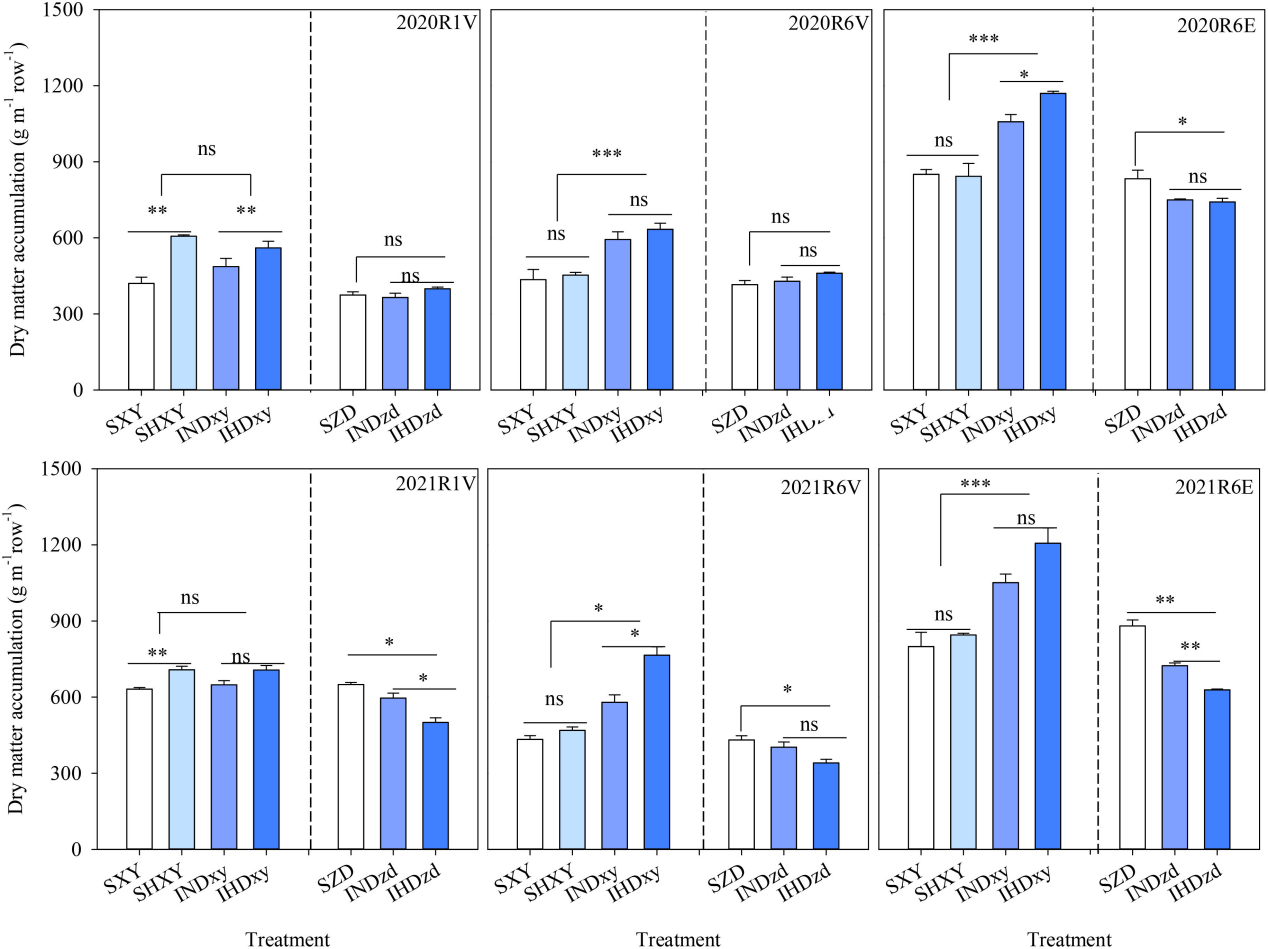
Effects of intercropping of tall- and short-stature maize cultivars on dry matter accumulation at silking (R1) and maturity (R6). V indicates vegetative organs (leaf and stem), and E indicates ear (cob + kernels + bract). SXY, sole XY335; SZD, sole ZD958; SHXY, sole XY335 with high density; IND, intercropping of ZD958 and XY335 under normal density; IHD, intercropping of normal density ZD958 and high density XY335. ns means no significant difference between cropping patterns or planting densities; * and ** indicate significant differences at *p* < 0.05 and *p* < 0.01, respectively.

Compared with sole XY, intercropping did not affect the dry matter accumulation at R1 but significantly increased the dry matter accumulation (both of vegetative and ear) at R6 over 2 years. The dry matter accumulation of ZD was obviously decreased in intercropping compared with sole ZD in 2021 (**Figure 7**). For sole XY, increasing the planting density significantly increased the dry matter weight at R1 but did not affect that at R6. For intercropped XY, dry matter weight was significantly increased under high density in 2020, but not in lodging 2021. Increasing the density of tall-stalked XY in intercropping markedly decreased the vegetative and ear biomass of intercropped ZD at R1 and R6 in 2021.

### 3.4 Bending strength and rind penetration strength

**Figure 8:**
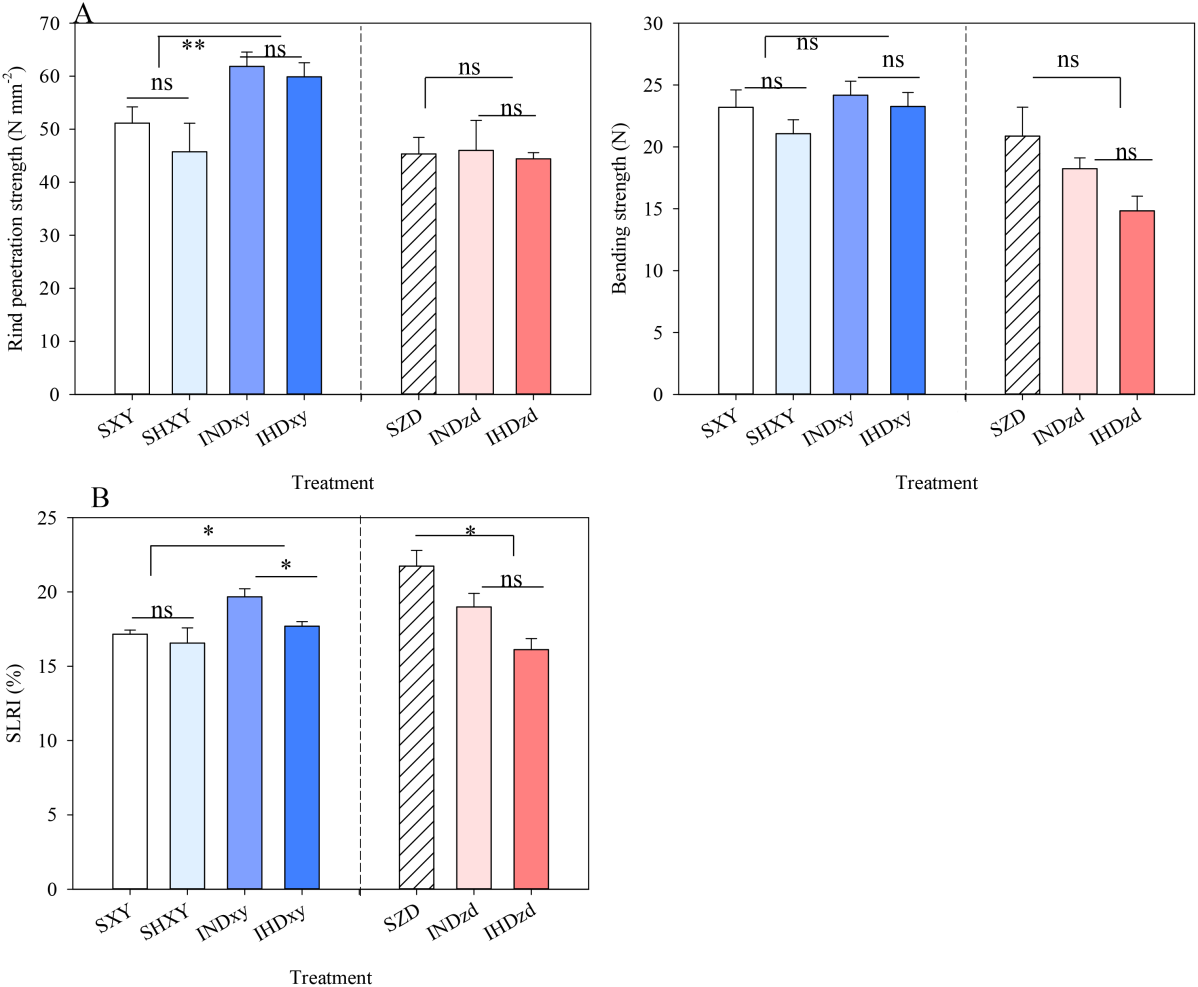
Effects of intercropping of tall- and short-stature maize cultivars on stem breaking strength and rind penetration strength **(A)** and stalk lodging-resistant index (SLRI) in 2021. SXY, sole XY335; SZD, sole ZD958; SHXY, sole XY335 with high density; IND, intercropping of ZD958 and XY335 under normal density; IHD, intercropping of normal density ZD958 and high density XY335. ns means no significant difference between cropping patterns or planting densities; * and ** indicate significant differences at *p* < 0.05 and *p* < 0.01, respectively.

Compared with sole XY, the rind penetration strength and stalk lodging-resistant index (SLRI) were significantly increased in intercropping (**Figure 8**). Therefore, no lodging was observed for intercropped XY; however, the lodging rate of sole XY was 4.3% and 22.0% under normal and high density conditions, respectively (**Supplementary Table S1**, **Supplementary Figure S4**). Conversely, intercropping had no significant effect on rind penetration strength and bending strength of ZD but significantly reduced the SLRI (**Figure 8**). It is worth noting that in the two growing seasons, lodging did not happen for ZD in all cropping patterns. Increasing the planting density decreased the rind penetration strength and bending strength of XY regardless of cropping pattern. Especially the SLRI of intercropped XY under high density was significantly lower than in normal density. High density XY in intercropping did not significantly affect the lodging-related indexes of ZD.

### 3.5 Soluble carbohydrate and starch

**Figure 9:**
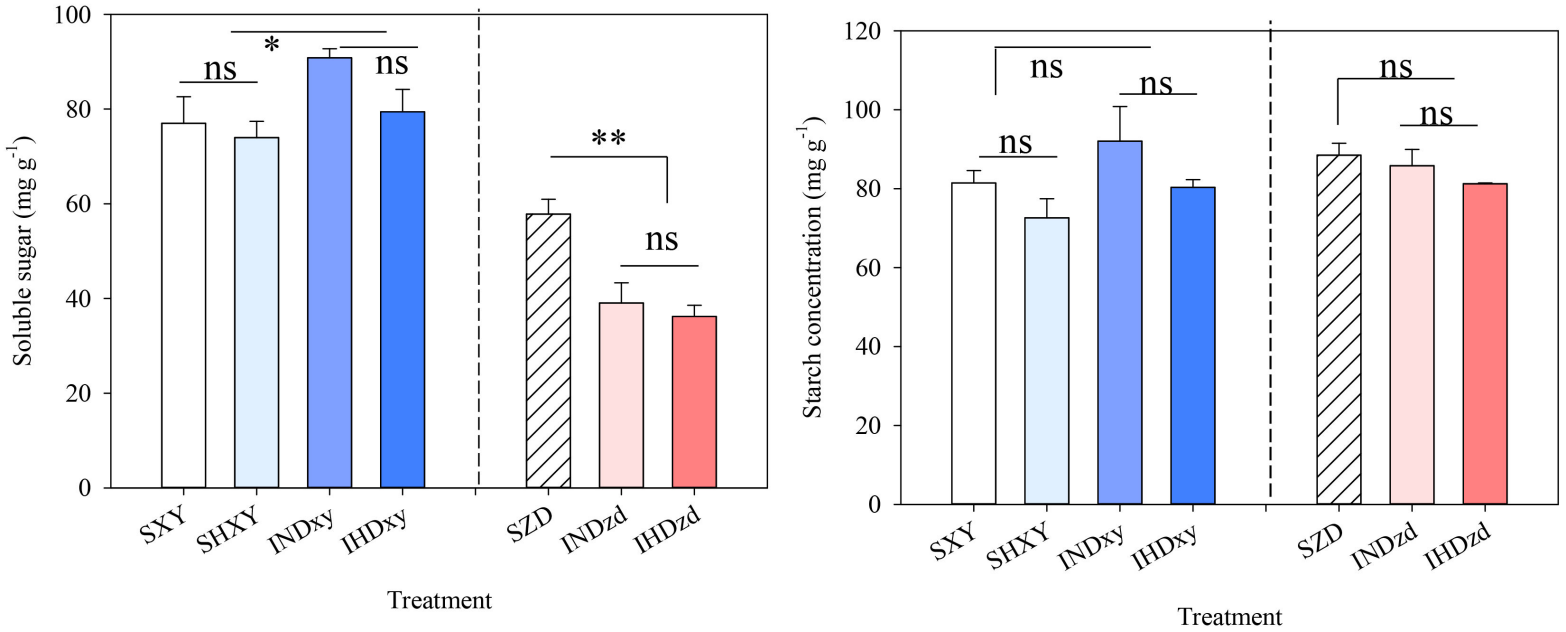
Effects of intercropping of tall- and short-stature maize cultivars on stem soluble sugar and starch concentration in 2021. SXY, sole XY335; SZD, sole ZD958; SHXY, sole XY335 with high density; IND, intercropping of ZD958 and XY335 under normal density; IHD, intercropping of normal density ZD958 and high density XY335. ns means no significant difference between cropping patterns or planting densities; * and ** indicate significant differences at *p* < 0.05 and *p* < 0.01, respectively.

Soluble carbohydrate and starch in stem were evaluated in 2021. Cultivar intercropping significantly increased the soluble sugar of tall-stalked XY by 12.8% compared to sole XY but dramatically decreased that of short-stalked ZD by 34.9% (**Figure 9**). Cropping pattern and plant density had no significant effect on the starch content of the two cultivars.

### 3.6 SPAD value, SOD activity, and MDA content

**Figure 10:**
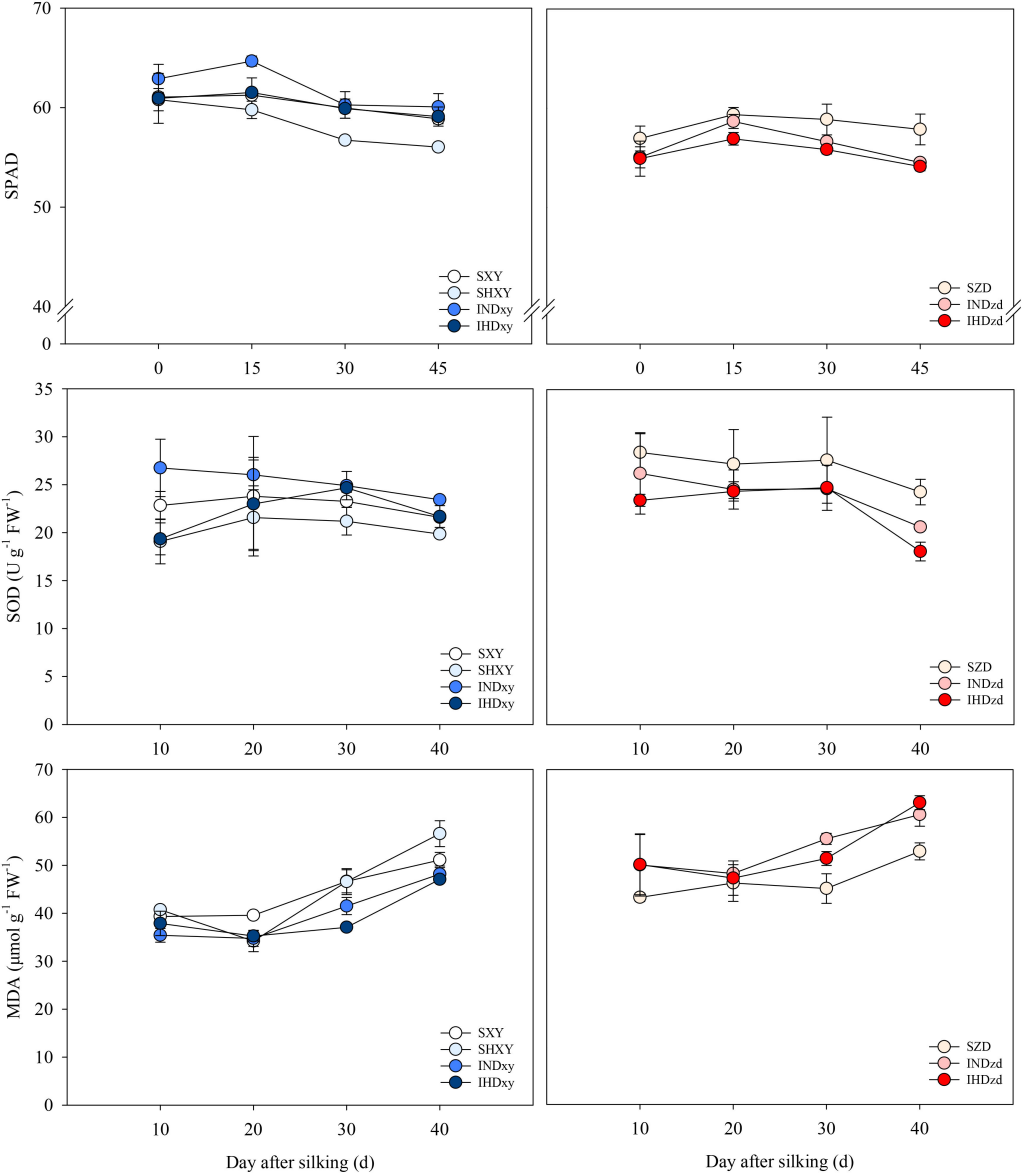
Effects of intercropping of tall- and short-stature maize cultivars on SPAD value, superoxide dismutase (SOD) activity, and malondialdehyde (MDA) content of ear leaf in 2021. SXY, sole XY335; SZD, sole ZD958; SHXY, sole XY335 with high density; IND, intercropping of ZD958 and XY335 under normal density; IHD, intercropping of normal density ZD958 and high density XY335.

Intercropping had positive effects on the SPAD of XY but had negative effects on that of ZD. Regardless of planting pattern, the SPAD of XY was decreased under high planting density. The SPAD of intercropped ZD was averagely 4.2% lower than sole ZD. The averaged SOD activity of intercropped XY was 7.3% and 8.6% higher than sole XY under normal and high planting density, respectively. However, the averaged SOD activity of intercropped ZD was 7.2% and 12.5% lower than sole ZD when intercropped with the normal and high density of XY, respectively. Intercropping decreased the MDA content of XY but increased that of ZD. The highest MDA content was recorded on SHXY and IHDzd, respectively (**Figure 10**).

### 3.7 Principal component analysis

**Figure 11:**
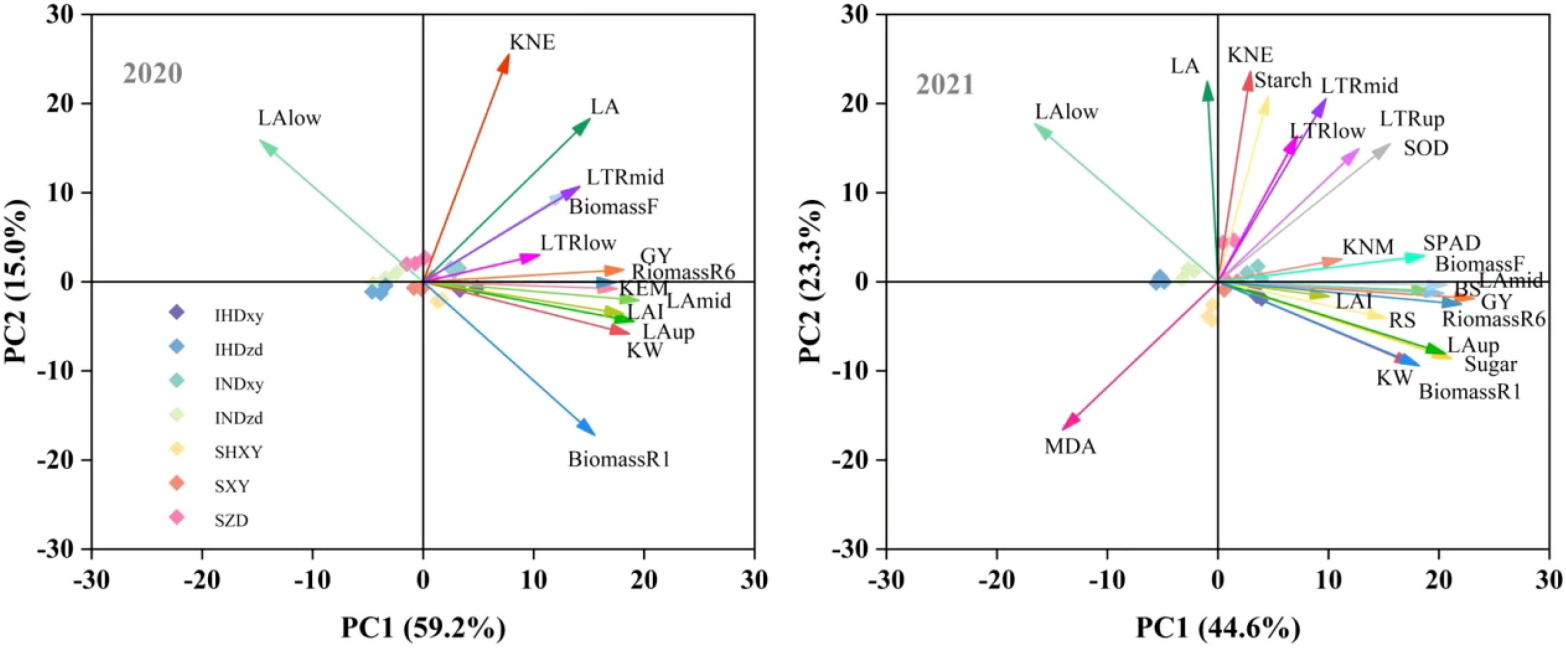
Principal component analysis of yield components, LAI, LTR, biomass. GY, grain yield; KNE, kernel number per ear; KNM, kernel number per square meter; LA, leaf area; LAup, LAmid, and LAlow indicate upper LA, middle LA, and lower LA, respectively; LTR, light transmission ratio; LTRup, LTRmid, and LTRlow indicate upper LTR, middle LTR, and lower LTR, respectively; BiomassR1, BiomassR6, and BiomassF indicate accumulated biomass at silking, at maturity, and during total filling stage; BS and RS mean bending strength and rind penetration strength.

The principal component analysis (PCA) of relevant indicators of yield, biomass, physiology, and lodging resistance in 2020 and 2021 are depicted in **Figure 11**. In 2020 and 2021, PC1 accounted for 59.2% and 44.6% of the variance between the variables, while PC2 explained 15.0% and 23.3% of the variability, respectively. A smaller acute angle represented a stronger correlation between variables. This indicated that the upper LA, middle LA, upper LTR, biomass, stem soluble sugar, SOD, and SPAD were closely associated with grain yield, while MDA was in the opposite direction to grain yield. Additionally, the LA of lower layer and the total LA were not closely related to grain yield (**Figure 11**).

## 4 Discussion

### 4.1 Intercropping tall- and short-stature maize cultivars did not result in yield penalty under high density

Considerable variations were observed in cultivar intercropping or mixture experiments. Numerous studies have demonstrated that cultivar intercropping or mixtures have great yield advantage, especially under adverse conditions (Fang et al., 2014; Li et al., 2023b). However, disadvantageous effects of cultivar intercropping or mixtures on yield had also been reported (Lithourgidis et al., 2011). Interestingly, mixtures designed with consideration of height differences exhibited overyielding of 2.8% compared to those without height consideration (Borg et al., 2018). In this study, tall- and short-stature maize intercropping did not show a significant yield advantage compared with monoculture. This was consistent with the results of maize cultivar mixtures, in which crude protein in grain was significantly increased but not for grain yield (Su et al., 2024). Moreover, a meta-analysis indicated that grain yields of intercropping or mixtures of cereal crop cultivars were only 2% to 3% higher than the pure stands (Borg et al., 2018; Kiær et al., 2009; Reiss and Drinkwater, 2018). Burton and Kemanian (2022) have proved that alternate low- and high-density rows could optimize the economic output and yield of maize. However, increasing the density of the tall component resulted in a slight yield increase compared to normal density intercropping in this study.

In our study, the plant height of XY exceeded that of ZD; thus, XY was considered as a dominant component and ZD as an inferior one. This observation is supported by the fact that the pLER of XY exceeded 0.5, while the partial LER of ZD fell below 0.5, indicating that ZD suffered competition from the taller one (**Figure 2**). This finding is in line with the research by Wang et al. (2017). The yield advantage of XY counteracted the yield loss of ZD, highlighting a compensatory effect rather than complementary effect in this cultivation. Moreover, increasing the density of tall-stalked maize intensified the competition between tall and short maize cultivars. The greater yield decline of ZD diminished the yield advantage of the intercropping system (**Figure 1**).

### 4.2 Tall-stature maize intercepted more light and leaf senesced slowly in intercropping, whereas short-stature maize was opposite

Intercropping leads to canopy heterogeneity and alters the microclimate, particularly light distribution, which, in turn, leads to spatial niche differentiation (Jurik and Van, 2004). The increased productivity of intercropping compared to monocultures often stems from enhanced light capture, more efficient light utilization, or a combination of both (Awal et al., 2006; Bedoussac and Justes, 2010). Significant height disparities can create competition for light and nutrients (Wang et al., 2021a). In our study, the upper layer leaf area of tall-stalked XY in intercropping was increased. The leaves located above the ear are a primary source of carbohydrates that contribute to the grain yield of maize (Li et al., 2024). The results of PCA indicated that the upper and middle leaf areas were closely related to grain yield. In contrast, the basal leaf area was far from yield (**Figure 11**). The plant architecture of ZD is characterized by a smaller leaf area in the upper and middle positions and a larger leaf area at the bottom (**Figure 5**). Intercropping further decreased the upper and middle leaf areas of ZD. In intercropping, the spatial distribution of leaf area is closely related to light interception (Umesh et al., 2023). Consequently, the increased upper and middle leaf areas of XY in intercropping intercepted more light, which decreased the radiation that reached the bottom of ZD, particularly when ZD was intercropped with high density XY (**Figure 6**). Yang et al. (2014) also reported that increasing the proportion of the taller component in the mixture decreased the light capture of the shorter one, consequently lowering the rate of photosynthesis in the shorter plants. Decreased leaf area (source) combined with low light interception finally caused the yield decline of ZD in intercropping, but taller XY was opposite. These findings aligned with previous research, which indicated that taller components intercept more light in intercropping, resulting in higher yields for the taller plants and lower yields for the shorter ones (Khalifa and Qualset, 1974).

**Figure 12:**
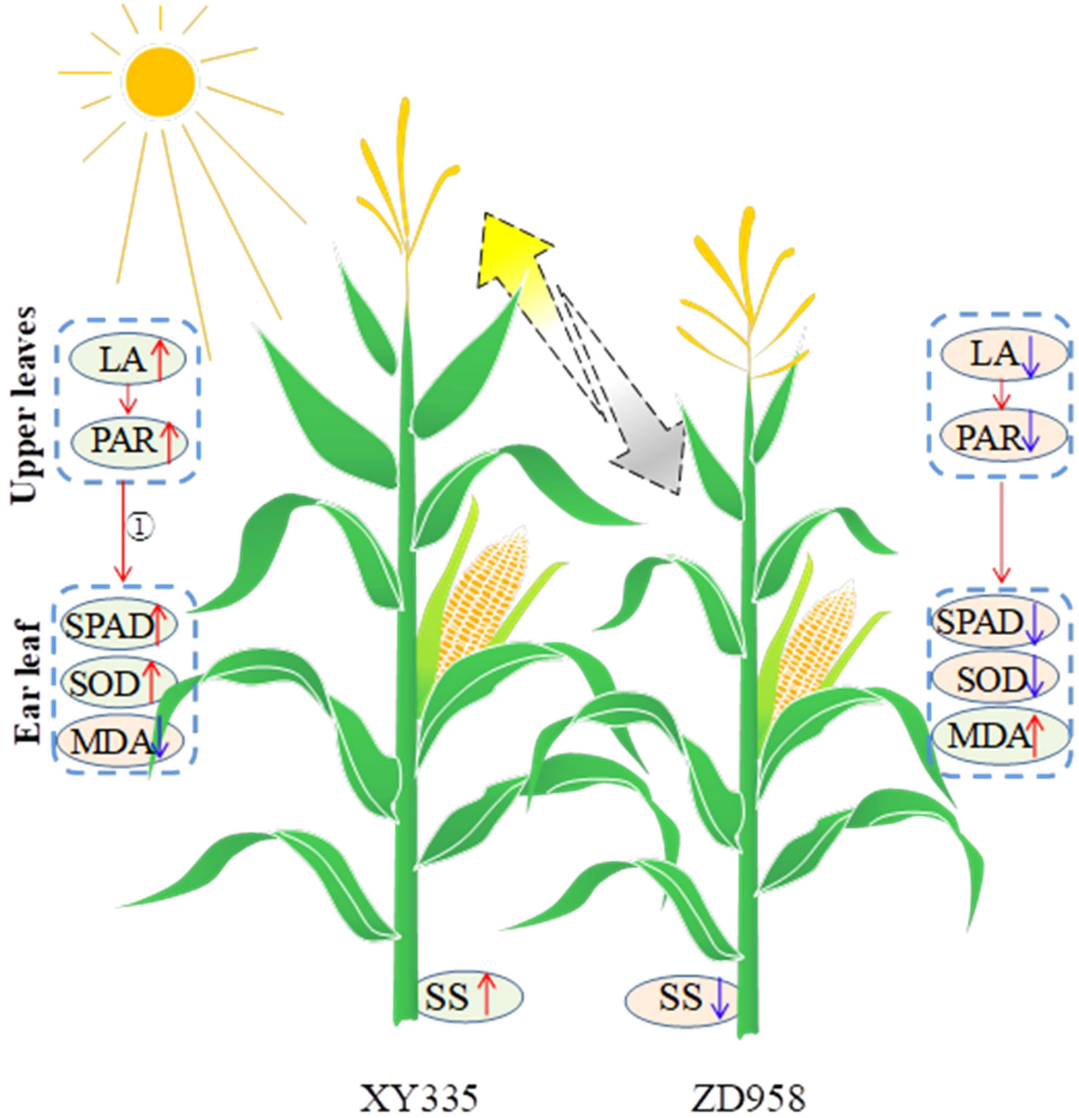
Graphical representation of changes in leaf area, light transmittance, SPAD, SOD, MDA, and soluble sugar (SS) in maize plants as affected by intercropping. The yellow and gray arrows show the good light and shade conditions. The red and blue arrows represent increase and decrease, respectively. ① refers to previous references (Deng et al., 2024; Feng et al., 2020).

Additionally, shade stress caused by taller plants impacts the shorter crop’s growth specifically during the reproductive stage, which leads to premature senescence of the shorter one in an intercropping system (Deng et al., 2024; Feng et al., 2020). Maize exhibited sensitivity to shade stress, particularly during the reproductive stage (Gao et al., 2020). As mentioned above, tall XY resulted in lower radiation interception of intercropped ZD, that is to say, intercropped ZD suffered shade stress from taller XY. Our study also showed that intercropped ZD displayed significant leaf senescence, but intercropped XY did not (**Figure 10**). The PCA indicated that the SOD activity and the SPAD value were close to grain yield. In addition, the light competition between tall- and short-stature maize cultivars led to a decreased stem soluble sugar content of ZD, consequently reducing the kernel number and grain yield of ZD. Based on the abovementioned results and discussion, a schematic diagram illustrating the impact of intercropping tall and short maize varieties on yield was drawn (**Figure 12**). Tall XY in intercropping showed a higher leaf area, thus intercepting more light. Accordingly, the soluble sugar availability in the stem was increased, and the leaf senescence of XY was mitigated. In contrast, the upper leaf area and PAR of short ZD in intercropping was reduced as caused by the shade from neighbor XY, which reduced the soluble sugar availability and accelerated leaf senescence. The ratio of border rows to total row number is a critical factor influencing light utilization, crop growth, yields, and yield components (Zhu et al., 2016). A higher proportion of border rows in intercropping provides a greater advantage in radiation capture due to border-row effects, e.g., increased direct radiation from the side of strip (van Oort et al., 2020; Zhu et al., 2016). In our study, a row configuration of 2:2 was implemented, meaning that both rows of each maize cultivar were designated as border rows. Consequently, taller XY benefited from the advantages of border rows, while shorter ZD suffered. This led to the growth inhibition of short-stature maize under low light conditions. We suggested that utilizing shade-tolerant cultivars or planting short maize in wider strips with a reduced proportion of border rows may enhance the positive effects of light for taller maize and mitigate the negative impacts on shorter maize. Furthermore, increasing the distance between two cultivar strips can also optimize light conditions. A supplementary experiment conducted in 2021 demonstrated that the grain yield of an intercropping system with increasing distance (80 cm) between XY and ZD strips averagely increased by 8.7% compared to corresponding intercropping with equal row spacing (60 cm; **Supplementary Figure S5**).

### 4.3 Intercropping tall- and short-stature maize cultivars decreased lodging risk

Previous studies had shown that intercropping or mixtures of tall and short wheat genotypes can reduce the lodging rate and enhance the crop lodging resistance (Cai et al., 2019; Kong et al., 2022). Our results also indicated that tall XY335 and short ZD958 intercropping reduced the lodging rate, especially under high density. The lodging resistance of plants relies on the mechanical strength of stems, which is influenced by structural carbohydrates such as cellulose and lignin (Liu et al., 2021). The more photosynthates used for maintaining stem strength, the less carbohydrate was available for kernel growth (Tang et al., 2022; Zhang et al., 2023). A trade-off existed between yield and lodging resistance. In our study, intercropping improved the stem mechanical strength of tall XY335, which might suggest that more photosynthates were used for enhancing lodging resistance. Consequently, the compromise between grain yield and lodging-related traits might reduce the yield potential of XY335 in intercropping.

The accumulation of structural carbohydrates is also influenced by the canopy light distribution and photosynthesis (Jin et al., 2023). Increasing the planting density reduces light interception and the photosynthetic capacity of leaves in the middle and lower layers, leading to compromised stem quality and increased risk of lodging (Liu et al., 2023). In our study, the intercropped XY intercepted more light, resulting in improved sugar accumulation, thereby enhancing stem strength. Xue et al. (2016) also indicated that increased light intensity at the middle layer significantly enhanced the rind penetration strength of the stem. In addition to stem quality, both lateral and longitudinal physical support of lodging-resistant cultivar to a lodging-susceptible cultivar in intercropping or mixtures can enhance the lodging resistance and yield stability of composite populations (Cai et al., 2019). Additionally, ZD exhibited a higher ear height (bending strength was determined at ear position), leading to a lower bending strength compared to XY. In the field, ZD demonstrated superior lodging resistance than XY due to the fact that the plant height of ZD was obviously lower than that of XY. Thus, XY experienced frequent lodging and had a higher lodging rate than ZD.

For high density maize production, application of plant growth retardant, optimized water, and nitrogen management were used to prevent lodging. These increased labor costs and agricultural input. Intercropping did not increase any input but decreased the lodging risk. Moreover, maize cultivar intercropping can mitigate heat stress (Li et al., 2023b) and control rust disease (Wu et al., 2024). Accordingly, we concluded that maize cultivar intercropping was a green strategy to enhance maize lodging resistance without yield penalty or requiring additional inputs.

### 4.4 Limitation of this study

Plant height was not affected by intercropping, but lodging was avoided in an intercropping system, which suggested that stem strength was enhanced. Though the cellulose and lignin of stem was not measured here, Li et al. (2023a) had clearly shown that increased soluble sugar in the stem provided sufficient substance to synthesize cellulose and lignin, thereby increasing the stem strength. This study indicated that intercropped XY showed higher soluble sugar in the stem, which also demonstrated that stem strength was enhanced. Only two cultivars were used in this study; however, increasing the number of cultivars may increase the yield (Su et al., 2023). XY and ZD used in this study had other different characteristics besides plant height (e.g., growth rate, leaf area, photosynthesis). Further research involving a broader germplasm and many traits is really needed to decipher the cultivar intercropping effects before cultivars combination rules can be provided to farmers.

## 5 Conclusion

In summary, we concluded that intercropping maize with different plant heights mainly increases the lodging resistance of tall maize by optimizing the canopy light distribution, thereby enhancing the lodging resistance of the composite population. However, short-stature maize cultivar in intercropping suffered from shade stress, especially when it was intercropped with high density tall-stalked maize, leading to leaf senescence and a larger yield loss. A suitable combination of cultivars and optimized field management are needed to further improve the yield performance of compound populations.

## Data Availability

The raw data supporting the conclusions of this article will be made available by the authors, without undue reservation.

## Supplementary material

Supplementary Figure 1 Average temperature, rainfall, photosynthetically active radiation (PAR), and wind speed in each month during maize growing seasons at the experimental site in 2020 and 2021.

Supplementary Figure 2 A diagram of the field layout showing different planting patterns. SXY, sole XY335; SZD, sole ZD958; SHXY, sole XY335 with high density; IND, intercropping of ZD958 and XY335 under normal density; IHD, intercropping of normal density ZD958 and high density XY335.

Supplementary Figure 3 Effects of intercropping of tall- and short-stature maize cultivars on plant height and ear height. SXY, sole XY335; SZD, sole ZD958; SHXY, sole XY335 with high density; IND, intercropping of ZD958 and XY335 under normal density; IHD, intercropping of normal density ZD958 and high density XY335. ns means no significant difference between cropping patterns or planting densities; *, **, and *** indicate significant differences at *p* < 0.05, *p* < 0.01, and *p* < 0.001, respectively.

Supplementary Figure 4 Photos of field plots in 2021. SXY, sole XY335; SZD, sole ZD958; SHXY, sole XY335 with high density; IND, intercropping of ZD958 and XY335 under normal density; IHD, intercropping of normal density ZD958 and high density XY335.

Supplementary Figure 5 Effects of row spacing on maize yield in intercropping systems in supplementary experiment in 2021. IND, intercropping of ZD958 and XY335 under normal density; IHD, intercropping of normal density ZD958 and high density XY335. W means 80–40-cm row spacing (row spacing between two cultivar strips is 80 cm, and row spacing for each cultivar is 40 cm). Different lowercase letters indicate significance at 0.05 level.

Supplementary Table 1 Lodging percentage under sole cropping and intercropping. SXY, sole XY335; SZD, sole ZD958; SHXY, sole XY335 with high density; IND, intercropping of ZD958 and XY335 under normal density; IHD, intercropping of normal density ZD958 and high density XY335.

Supplementary Table 2 ANOVA results of year, density, intercropping, and their interactions on each index.
