# Supplementary material for: Intercropping of short- and tall-stature maize decreases lodging risk without yield penalty at high planting density
Source: Front Plant Sci. 2025 Apr 30;16:1570921. doi: 10.3389/fpls.2025.1570921 (PMC12075153; doi:10.3389/fpls.2025.1570921)
Supplement: Supplementary Figure 1 — Average temperature, rainfall, photosynthetically active radiation (PAR), and wind speed in each month during maize growing seasons at the experimental site in 2020 and 2021. [file SupplementaryFile1.docx]

Fig. S1

Average temperature, rainfall, photosynthetically active radiation (PAR), and wind speed in each month during maize growing seasons at the experimental site in 2020 and 2021.

Fig. S2

A diagram of the field layout showing different planting patterns. SXY, sole XY335; SZD, sole ZD958; SHXY, sole XY335 with high density; IND, intercropping of ZD958 and XY335 under normal density; IHD, intercropping of normal density ZD958 and high density XY335.

Fig. S3

Effects of intercropping of tall and short stature maize cultivars on plant height and ear height. SXY, sole XY335; SZD, sole ZD958; SHXY, sole XY335 with high density; IND, intercropping of ZD958 and XY335 under normal density; IHD, intercropping of normal density ZD958 and high density XY335. ns means no significant difference between cropping patterns or planting densities; *, **, and *** indicate significant differences at p<0.05, p<0.01 and p<0.001, respectively.

Fig. S4

The photos of field plots in 2021. SXY, sole XY335; SZD, sole ZD958; SHXY, sole XY335 with high density; IND, intercropping of ZD958 and XY335 under normal density; IHD, intercropping of normal density ZD958 and high density XY335.

Fig. S5

Effects of row spacing on maize yield in intercropping systems in supplementary experiment in 2021. IND, intercropping of ZD958 and XY335 under normal density; IHD, intercropping of normal density ZD958 and high density XY335. W means 80-40 cm row spacing (row spacing between two cultivars strip is 80 cm and row spacing for each cultivar is 40 cm). Different lowercase indicates significance at 0.05 level.

Table S1 Lodging percentage under sole cropping and intercropping. SXY, sole XY335; SZD, sole ZD958; SHXY, sole XY335 with high density; IND, intercropping of ZD958 and XY335 under normal density; IHD, intercropping of normal density ZD958 and high density XY335.

| Treatment | Lodging percentage (%) | |
| --- | --- | --- |
|  | 2020 | 2021 |
| SXY | 0 | 4.2b |
| SZD | 0 | 0c |
| SHXY | 0 | 22.0a |
| IND | 0 | 0c |
| IHD | 0 | 0c |

Table S2 The ANOVA results of

| Variety |  | Yield (g m^-1^ row^-1^) | TKW (g) | KNE | upLA | midLA | lowLA | LAI | Plant height | Ear height | R1V | R6V | R6E |
| --- | --- | --- | --- | --- | --- | --- | --- | --- | --- | --- | --- | --- | --- |
| XY | Year | 0.000 | 0.760 | 0.000 | 0.002 | 0.982 | 0.007 | 0.048 | 0.000 | 0.000 | 0.000 | 0.143 | 0.876 |
|  | Density | 0.005 | 0.106 | 0.002 | 0.128 | 0.525 | 0.046 | 0.003 | 0.392 | 0.366 | 0.000 | 0.011 | 0.027 |
|  | Intercropping | 0.000 | 0.915 | 0.002 | 0.000 | 0.123 | 0.970 | 0.089 | 0.377 | 0.933 | 0.482 | 0.000 | 0.000 |
|  | Y*D | 0.046 | 0.051 | 0.837 | 0.300 | 0.769 | 0.316 | 0.468 | 0.023 | 0.923 | 0.026 | 0.097 | 0.453 |
|  | Y*I | 0.183 | 0.113 | 0.717 | 0.506 | 0.393 | 0.448 | 0.144 | 0.472 | 0.090 | 0.940 | 0.274 | 0.520 |
|  | D*I | 0.079 | 0.592 | 0.198 | 0.334 | 0.365 | 0.884 | 0.614 | 0.362 | 0.888 | 0.021 | 0.092 | 0.081 |
|  | Y*D*I | 0.034 | 0.613 | 0.567 | 0.640 | 0.385 | 0.996 | 0.114 | 0.025 | 0.155 | 0.078 | 0.206 | 0.941 |
| ZD | Year | 0.000 | 0.000 | 0.000 | 0.000 | 0.005 | 0.000 | 0.000 | 0.000 | 0.000 | 0.000 | 0.004 | 0.332 |
|  | Density | 0.115 | 0.963 | 0.358 | 0.003 | 0.005 | 0.992 | 0.209 | 0.077 | 0.051 | 0.059 | 0.321 | 0.092 |
|  | Intercropping | 0.000 | 0.001 | 0.000 | 0.042 | 0.010 | 0.276 | 0.059 | 0.989 | 0.982 | 0.056 | 0.605 | 0.003 |
|  | Y*D | 0.105 | 0.338 | 0.591 | 0.000 | 0.008 | 0.022 | 0.209 | 0.674 | 0.134 | 0.001 | 0.009 | 0.247 |
|  | Y*I | 0.025 | 0.013 | 0.200 | 0.029 | 0.026 | 0.443 | 0.610 | 0.899 | 0.487 | 0.171 | 0.186 | 0.263 |
|  | D*I | － | － | － | － | － | － | － | － | － | － | － | － |
|  | Y*D*I | － | － | － | － | － | － | － | － | － | － | － | － |
